# Segmental Testicular Infarction Due to Arterial Atheroma: A Case Report

**DOI:** 10.7759/cureus.30506

**Published:** 2022-10-20

**Authors:** Edward J M Hart, Samuel S Folkard

**Affiliations:** 1 Urology, University Hospitals Sussex NHS Foundation Trust, Brighton, GBR; 2 Urology, Maidstone and Tunbridge Wells NHS Trust, Maidstone, GBR

**Keywords:** orchidectomy, atheroma, testicular mass, scrotal pain, segmental testicular infarction

## Abstract

Segmental testicular infarction is an uncommon condition, of which the majority of cases are idiopathic. Cases associated with atherosclerotic disease are extremely rare, with only two other cases reported in the literature to our knowledge. We report the case of a 71-year-old man who presented with left testicular pain and a mass. Ultrasound imaging confirmed an upper pole left testicular mass, and he subsequently underwent radical inguinal orchidectomy for presumed testicular malignancy. Histological analysis revealed a segmental testicular infarction related to arterial atheroma. Segmental testicular infarction can mimic other pathologies, such as testicular torsion or cancer, resulting in misdiagnosis and potentially unnecessary surgery. Increased awareness of the risk factors and clinical features may help clinicians identify and appropriately manage this uncommon pathology.

## Introduction

Segmental testicular infarction is an uncommon cause for acute scrotal pain or scrotal mass, representing less than 1% of these patients [1], and is therefore often overlooked as a differential diagnosis. In the majority of cases, the underlying cause is unknown, but it has been associated with a number of pathologies, including epididymo-orchitis, vasculitides, and sickle cell disease [2-6]. Segmental testicular infarction due to atherosclerotic disease is very rare, with only two other reported cases in the literature to our knowledge [7,8]. We report the case of a 71-year-old man who presented with left testicular pain and mass, found to be caused by an arterial atheroma but mistaken for a testicular tumour.

## Case presentation

A 71-year-old male, known smoker, presented to his general practitioner with subacute left scrotal pain, ongoing for approximately three months. He had a significant past medical history of peripheral vascular disease with right critical limb ischaemia requiring recent right common femoral endarterectomy and femoropopliteal atherectomy, bilateral deep vein thromboses (DVT) with post-phlebitic syndrome, and chronic obstructive pulmonary disease (COPD). He was taking appropriate secondary preventative medications for atherosclerotic disease, in addition to apixaban.

**Figure 1:**
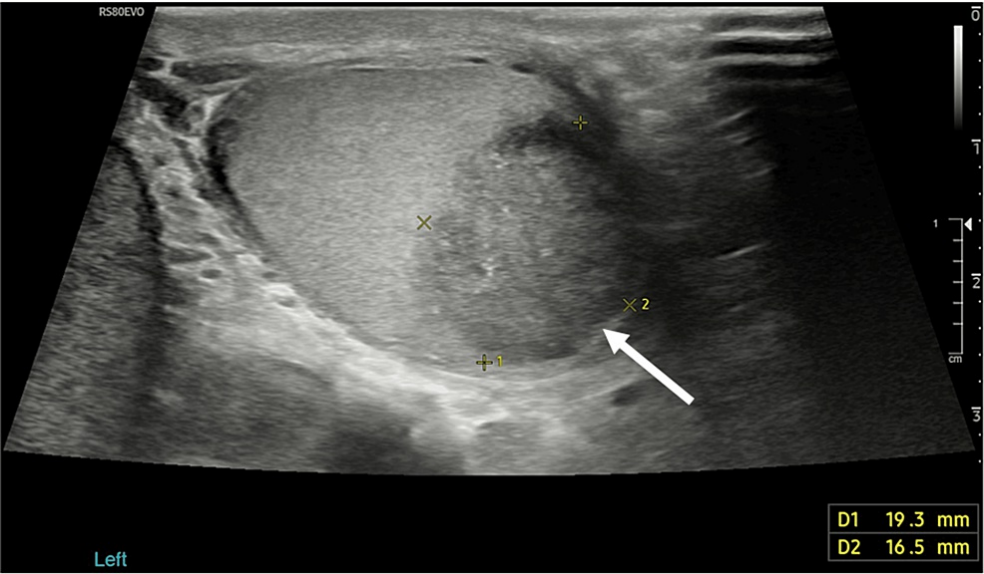
Ultrasound testes revealed a 19 mm x 17 mm left upper pole testicular mass with hyperechoic flecks

**Figure 2:**
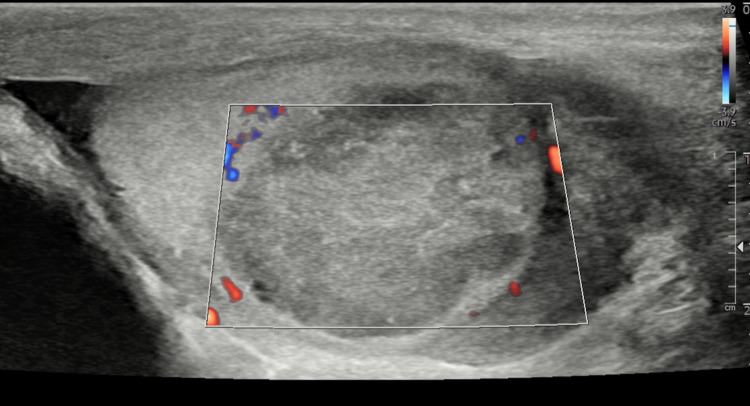
Ultrasound testes of the avascular left testicular lesion demonstrated by absent colour Doppler flow

He had no history of urinary tract or sexually transmitted infections and had a negative urinalysis and urine culture. He denied previous scrotal trauma, haematuria, or fever. Initial clinical examination findings were a hard mass in the upper pole of the left testicle, with a normal right testicle apart from a small epididymal cyst. Testicular tumour markers were within normal ranges (alpha-fetoprotein (AFP)=3 ng/mL; human chorionic gonadotropin (HCG)<2 IU/L; lactate dehydrogenase (LDH)=214 units/L), and no significant abnormalities were detected on full blood count, renal function, or C-reactive protein (CRP). An ultrasound was performed which revealed a 19 mm x 17 mm left upper pole testicular mass, with hyperechoic flecks and no vascularity demonstrated (Figures 1, 2). He was subsequently referred to our service.

A left testicular malignancy was suspected, including lymphoma, teratoma or seminoma. Following counselling, the patient opted for radical surgery over active monitoring. He underwent a left radical orchidectomy via an inguinal incision. Intra-operative findings were a firm lesion at the upper pole of the left testis, suggestive of a testicular tumour. The operation was uncomplicated, and the patient was discharged the same day. He was reviewed in the clinic one month post-operatively and had made a full recovery.

A macroscopic examination of the specimen revealed a presumed solid tumour located in the upper pole, with the tunica vaginalis moving freely over the tumour. Microscopic examination identified the lesion as an area of segmental infarction with necrotic ghost tubules and vessels containing fibrin. Some of the subcapsular arteries were severely atheromatous with cholesterol clefts within one of the plaques. The overall appearance was of an ischaemic testicular infarct-related to arterial atheroma, with no evidence of malignancy.

## Discussion

Global testicular infarction is common and, in most cases, is secondary to testicular torsion [9]. In contrast, segmental testicular infarction is a rare diagnosis in the context of scrotal pain, with only case reports and limited case series previously documented [1,10]. Previous studies have shown that 0.3-3.5% of patients who undergo ultrasound imaging for acute scrotal pain are diagnosed with segmental testicular infarction [1,10]. Moreover, the incidence of segmental testicular infarction associated with arterial atheroma or embolism is even more uncommon, with only two other cases reported in the literature to our knowledge [7,8].

It is hypothesised that segmental areas of the testis are functional end organs, explaining why arterial or venous obstruction can lead to segmental infarctions. The testicle is supplied by three arteries: the testicular artery, the deferens artery, and the cremasteric artery. The primary blood supply is from the testicular artery, originating from the abdominal aorta, which subsequently divides into an anterior branch supplying the superior aspect, and a posterior branch supplying more of the inferior aspect. The posterior branch receives collateral blood flow from the deferens and cremasteric arteries. This may explain the increased incidence of superior segmental testicular infarcts [1,11], as was the case in our patient, given the lack of collateral blood supply.

In most cases of segmental testicular infarction, an underlying cause is not identified [10]. However, a number of predisposing factors have been reported, including epididymo-orchitis, trauma, post-surgery, partial torsion, hypercoagulable states, and, in this case, atheromatous disease [2,3,5,7,8,12,13]. The patient had a number of risk factors for atheromatous embolism; he was a current smoker, taking anticoagulant medication, and had a CT angiogram confirmed atherosclerotic vascular disease involving his abdominal aorta and bilateral lower limbs, with high-grade right superficial femoral artery stenoses. In addition, the onset of his symptoms occurred shortly after an invasive endovascular procedure to treat an ischaemic right lower limb due to common femoral artery stenosis. Anatomically, a procedure involving manipulation of the aorta would pose a higher risk for atheromatous embolism causing an ischaemic testicular infarct, given the aortic origin of the testicular artery [14]. Nevertheless, an invasive procedure of this nature remains a significant risk factor.

Clinical diagnosis of segmental testicular infarction is often challenging due to its non-specific presentation with scrotal pain and/or testicular mass, often mimicking testicular torsion or tumour [15]. Basic investigations include urine dip, routine blood tests and tumour markers (LDH, AFP, and HCG), which were all normal in this case. Scrotal ultrasound is first-line imaging in patients with scrotal pain or mass. The typical sonographic appearance of a segmental testicular infarct is a hypoechoic round or wedge-shaped area, with a well-defined border and most often avascular on Doppler [7]. This can aid in distinguishing infarcts from testicular tumours, which usually demonstrate normal or increased vascularity. However, sometimes testicular tumours may also appear avascular, making a safe distinction between the two difficult, especially in more rounded lesions, as was found in our patient [7,16].

In cases of diagnostic uncertainty, a period of close observation with serial ultrasound and tumour markers may be a reasonable approach, especially in young patients and those wishing to preserve fertility [1,7,17]. However, caution is needed in patients with concerning features such as elevated tumour markers. Alternatively, MRI imaging can help delineate the two pathologies, and prevent the need for operative intervention [8,18].

If clinical or radiological features fail to confirm the diagnosis, histological confirmation is required. In this case, the patient preferred the certainty of surgery, and hence a radical inguinal orchidectomy was performed. For patients opting for radical surgery, it is imperative to counsel them on the possibility of benign histological findings, particularly for older patients presenting outside the typical age group for testicular tumours [19]. Several cases of testis-sparing approaches have been reported, such as partial orchidectomy, or the use of peri-operative frozen section examination [1,12]. The latter has been shown to be effective for gaining a histological diagnosis and limiting the rate of orchidectomy for non-malignant lesions, whilst also maintaining oncological safety [19,20].

## Conclusions

In conclusion, segmental testicular infarction remains a diagnostic challenge for the clinician due to its low incidence and non-specific presentation that mimics other scrotal pathologies. It is an important differential diagnosis to consider, as recognition can avoid unnecessary orchidectomy. Segmental testicular infarction secondary to arterial atheroma is extremely rare, however, it may be that many previous cases have been misdiagnosed as idiopathic when histological analysis has not been acquired. In patients with known atherosclerotic disease or risk factors, it should be considered. Ultrasound can aid in distinguishing between segmental infarction and tumour; however, histology is often required to confirm the diagnosis.
